# External genital features and normative values for anogenital distances and penile length at birth in a healthy Italian population: data from the LIFE-MILCH project

**DOI:** 10.3389/fendo.2026.1838976

**Published:** 2026-08-06

**Authors:** Beatrice Righi, Annalisa Pelosi, Francesca Alberghi, Marta Fontana, Emanuela Davolio, Cecilia Rotteglia, Chiara Sartori, M. Maddalena Brambilla, Chiara Petrolini, Serafina Perrone, Anna-Mariia Shulhai, Angelica Dessì, Silvia Petza, Roberta Pintus, Alessandro De Fanti, Giancarlo Gargano, Anna Maria Papini, Vassilios Fanos, Paola Palanza, Maria Elisabeth Street

**Affiliations:** 1 Unit of Paediatrics, Department of Mother and Child, Azienda Unità Sanitaria Locale (USL)-IRCCS di Reggio Emilia, Reggio Emilia, Italy; 2 Department of Medicine and Surgery, University of Parma, Parma, Italy; 3 Unit of Obstetrics and Gynecology, Azienda Unità Sanitaria Locale (USL)-IRCCS of Reggio Emilia, Reggio Emilia, Italy; 4 Neonatology Unit, Department of Medicine and Surgery, University of Parma, Pietro Barilla Children’s Hospital, Parma, Italy; 5 Neonatal Intensive Care Unit, Department of Surgical Sciences, University of Cagliari, Azienda Ospedaliero Universitaria (AOU) Cagliari, Cagliari, Italy; 6 Department of Mother and Child, Neonatal Intensive Care Unit, Azienda Unità Sanitaria Locale (USL)-IRCCS di Reggio Emilia, Reggio Emilia, Italy; 7 Department of Chemistry “Ugo Schiff”, University of Florence, Sesto Fiorentino, Italy; 8 Department of Medicine and Surgery, University of Cagliari, Cagliari, Italy; 9 Unit of Paediatrics, University Hospital of Parma, Parma, Italy

**Keywords:** anogenital distance, newborn, normative values, penile length, references

## Abstract

**Introduction:**

Anogenital distance (AGD) and penile length (PL) are considered as surrogate biomarkers of fetal androgen action and are used to study the impact of prenatal exposure to endocrine disrupting chemicals. Data for newborn sex-specific references of both AGD and PL are currently scarce for the Italian population. This study aimed to provide normative data in a large group of Italian newborns and to assess any associations with anthropometric parameters.

**Methods:**

Data were collected at birth in mother-infant dyads from April 2021 to September 2022. Four hundred and sixty-two/675 neonates at term of uncomplicated pregnancies were enrolled from three different Italian Hospitals. Newborns were divided in 5 subgroups according to weeks of gestational age(GA):1(37-37 + 6d w),2(38-38 + 6d w),3(39-39 + 6d w),4(40- 40 + 6d w),5(> 41w) and in 4 sub-groups according to birth weight (BW): A(2501–3000 g), B(3001–3500 g), C(3501–4000 g), D (>4001 g). Trained staff measured AGD and PL in newborns with an analogical caliper using the TIDES method. Anogenital distances (AGDs) considered were: ano-scrotal (AGD_AS_) and ano-penile (AGD_AP_) in males, ano-fourchette (AGD_AF_) and ano-clitoral (AGD_AC_)in females. Correlations among AGDs and anthropometric measures were evaluated by Pearson correlation analysis. P<0.05 was significant. Data were expressed as mean± standard deviation.

**Results:**

Overall, in males AGDs were longer than in females. Considering the 5 subgroups for GA, AGD_AP_ was significantly longer than AGD_AC_ in neonates born at and after 40 weeks(p<0.05) whereas AGD_AS_ was significantly longer than AGD_AF_ in neonates born at and after 39 weeks(p<0.05). AGD_AS_ increased with increasing GA. Considering AGDs according to BW, AGD_AP_ was significantly longer than AGD_AC_ in newborns with a BW between 3001 and 4000 g(p<0.05), whereas AGD_AS_ was significantly longer than AGD_AF_ in newborns with a BW between 2501 g and 4000 g(p<0.05). Mean PL of male newborns (N = 208) was 24.4 ± 6.83 mm and increased with GA. In the correlation analysis a positive significant correlation was found between AGD_AS_ and PL (r=0.576, p<0.001).

**Conclusion:**

This study provided measurements and reference values for AGD and PL, based also on GA and BW, in a large group of newborns, supplying a valid well-reproducible instrument for clinical practice and the basis for further studies.

## Introduction

During embryogenesis, after the indifferent stage of external genitalia, the ventral migration of the genital swelling, urethral folds and possibly the genital tubercle evolve under the influence of androgens determining sexual dimorphism of anogenital distance (AGD) (1–3). AGD, measured from the center of anus to the genital tubercle, is regarded as a sensitive marker of prenatal androgen action in animals (4). For example, in rodents, AGD is related to perineal growth and androgen-dependent caudal migration of the genital tubercle and is affected by exposure to anti-androgens during a critical phase of testis development known as the “masculinization programming window” (MPW) (2). The difference in AGD between males and females can be therefore explained by intrauterine androgen exposure that is responsible for the external masculinization of genitalia (3, 5).

Since AGD in early gestation could be considered a surrogate biomarker of *in utero* androgen action in both animals and humans, it has been found to be affected by exposure to compounds that cause disruption or deficiency of androgen action. Over time in fact, AGD measurements have been used to study the impact of prenatal exposure to agents that can affect the endocrine system (4), and has become a valuable measure for reproductive toxicity studies as stated in the US Environmental Protection Agency guidelines (6). Several studies have shown a reduction in AGD in association with exposure to certain groups of Endocrine Disrupting Chemicals (EDCs) during gestation, such as phthalates, bisphenol A and dioxins (2). Penile length (PL) is related also to androgen exposure (4).

AGD has, therefore, been associated with multiple human reproductive health outcomes (2). A shorter AGD in males has been described in association with undescended testis and hypospadias in newborns (7) and with prostate cancer risk and infertility in adult life whereas a longer AGD in females has been associated with polycystic ovary syndrome, menstrual cycle irregularities and endometriosis (2) and congenital adrenal hyperplasia (8).

In humans, due to the different structure of male and female external genitalia, different measurements of AGD are used for the same purposes (9). The AGD variants in males are: from the center of the anus to the anterior base of the penis, from the center of the anus to the posterior base of the penis, and from the center of the anus to the anterior base of the scrotum. The AGD variants in females are: from the center of the anus to the posterior commissure of the labia minora (fourchette), from the center of the anus to the clitoris and from the fourchette to the clitoris (9).

Previous studies have investigated AGD in humans, particularly in newborns and infants, showing some differences based on the methodology used and the population examined (1, 5, 6, 8–12). Furthermore, normative references depend on ethnic differences (4) and newborn sex-specific references of both AGD and penile length are currently scarce for the Italian population (13). This study aimed to provide normative data for both males and females in a large group of Italian newborns, collected within the European project-Programme EASME-GIE Life 2018- Environment and Health: Mother and Infants dyads: Lowering the impact of endocrine disrupting Chemicals in milk for a Healthy Life (LIFE-MILCH), and to assess any associations with neonatal and maternal parameters.

## Patients and methods

### Patients

Data were collected at birth in mothers and their newborns (14, 15).

The inclusion criteria of mother-infant dyads were: uncomplicated singleton pregnancies in healthy women, newborns born at term, availability to the 12 month-follow-up. The enrolment started in April 2021 and ended in September 2022. Mothers were enrolled at 36 weeks of gestational age (GA) assessed by last menstrual period. Preterm and small for gestational age (SGA) newborns were excluded from the study.

**Figure 1:**
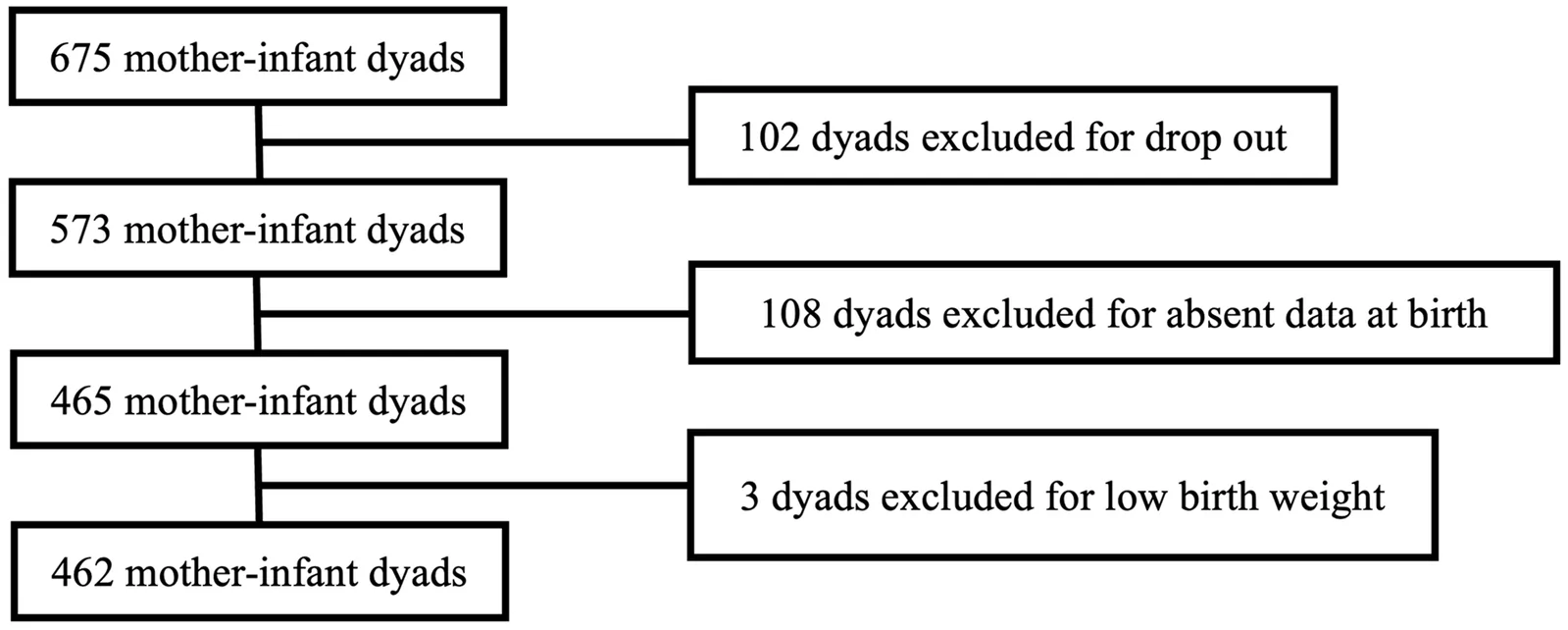
Consort diagram of the study.

Overall, 675 mothers and their newborns were screened and 462 mother-infant dyads were enrolled from three different Italian Hospitals (Parma, Reggio Emilia and Cagliari Hospitals) as represented in **Figure 1**. Among newborns, males were 253 and females were 209.

At recruitment, the body mass index (BMI) of the mothers at the beginning of pregnancy was calculated based on their height and weight. BMI was calculated as kg/m^2^ (16).

At birth, trained staff (a neonatologist and a pediatric endocrinologist) performed a clinical evaluation of the external genitalia, description of external genitalia (es: malformations, swelling, position of testicles), AGD measurements and, in males, measurement of PL. The volume of the testes in males was assessed using a standard Prader orchidometer. Length, weight, head circumference, sex and GA at birth were obtained by clinical records. Weight for length Z-score was calculated using a clinical calculator online according to WHO standards (17).

The main characteristics of the mothers and newborns are summarized in **Table 1**.

**Table 1:** Features of the mothers and newborns included in the study. Data are expressed as mean **±** SD.

Maternal data	Mean ± SD		
Age (y)	33.6 ± 4.18		
Height (cm)	164.9 ± 6.71		
Weight before pregnancy (kg)	62.3 ± 10.9		
Weight at the end of pregnancy (kg)	74.6 ± 11.9		
BMI before pregnancy (kg/m2)	22.8 ± 3.66		
Neonatal data	Mean ± SD	Mean ± SD	Mean ± SD
	Overall	Males	Females
GA (weeks)	39.7 ± 1.11	39.7 ± 1.17	39.6 ± 1.04
Weight (g)	3423 ± 409	3502 ± 415	3342 ± 386
Length (cm)	51.0 ± 1.99	51.4 ± 2.0	50.6 ± 1.89
Head circumference (cm)	34.5 ± 1.99	34.9 ± 1.38	34.2 ± 2.42
Weight-for-length Z-score	-0.493	-0.475 ± 1.37	-0.512 ± 1.13

### AGD and penile length measurements

**Figure 2:**
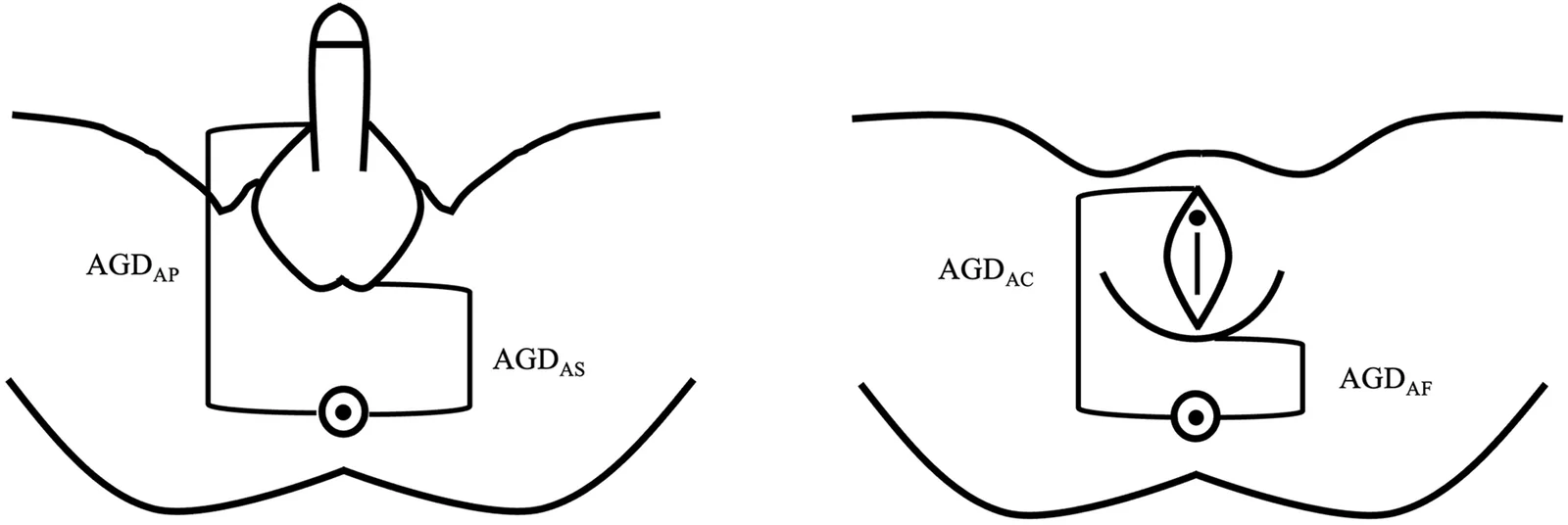
AGD measurements in males and females according to the TIDES method. In males (left) AGD_AP_: ano-penile, AGD_AS_: ano-scrotal. In females (right) AGD_AC_: ano-clitoral, AGD_AF_: ano-fourchette.

Within the first 72 hours of life, expert investigators measured anogenital distances (AGDs) using an analogical caliper, that was of identical model and resolution across all three study centers. Short AGDs represent the distance from the anus to the perineum–scrotal junction (ano-scrotal, AGD_AS_) in boys and from the anus to the fourchette (ano-fourchette, AGD_AF_) in girls. Similarly, long AGDs refers to the distance from the anus to the anterior insertion of the penis (ano-penile, AGD_AP_) in boys and from the anus to the clitoris (ano-clitoral, AGD_AC_) in girl as previously described by Fisher at al (6). and as shown in **Figure 2**.

Newborns were positioned according to TIDES method (8) placing the newborn supine with the lower half of the body exposed and the legs lifted in a frog-like posture (with a 60–90° angle from the torso at the hip) and knees pulled back towards to the shoulders of the operator holding the child.

PL was measured using an analogical caliper on the dorsal surface of the penis with the caliper against the pubis. A gentle traction was applied along the length of the penis to a point of increased resistance.

AGD and PL measures were performed once and reliably in a clinical research setting. Training videos or conducted in-person training and protocols were shared among the researchers.

### Statistical analysis

Newborns were subdivided by sex and in 5 sub-groups according to weeks of GA: 1(37-37 + 6d w), 2(38-38 + 6d w), 3(39-39 + 6d w), 4(40- 40 + 6d w), 5(> 41 w) and in 4 sub-groups according to birth weight (BW): A (2501–3000 g), B (3001–3500 g), C (3501–4000 g), D (>4001 g). AGDs and PL were expressed in millimeters (mm).

Descriptive statistics (means, standard deviations, percentile ranks) were used to describe the sample and identify the percentile distribution of the AGDs. Penile length and AGDs percentiles were empirically calculated, applying a linear interpolation, as all distributions were too skewed to fit a normal model.

Linear models were used to estimate the significance of differences between groups (analysis of variance, ANOVA), and Pearson’s r correlation coefficients were used to assess the existence of linear relationships between continuous variables. In all analyses, the threshold for significance was alpha = 0.05, applying the Holm correction for multiple correlations to reduce the inflation of Type I errors.

Data were expressed as mean ± standard deviation (SD). Interexaminer variation was evaluated using intraclass correlation coefficients (ICCs), calculated with repeated measure, nested two-way mixed-effects models. All analyses were performed with R software (v. 4.4.2) (18).

## Results

### General features of external genitalia

Two hundred and forty-six/253 (97%) male newborns had palpable testes, all with prepubertal volumes. Two boys presented a bilateral testicular volume of 3 ml with no abnormal endocrine findings at follow-up. Five (2%) newborns had one unpalpable testis, 2 (1%) presented bilateral cryptorchidism at birth. Hydrocele was described in 81 (32%) newborns: mild in 74 (91%), moderate in 5 (6%), severe in 2 (3%). Two newborns presented with coronal hypospadias.

Among females, 6 (3%) presented swelling of the labia majora, 1 labia majora hypertrophy, 1 mild clitoral hypertrophy, 1 mild clitoral hypertrophy and swelling of the labia majora. Eleven (5%) presented with genital crisis few days after birth.

Overall, 252 newborns (55%) presented bilateral enlarged breast buds at birth, whereas 18 (4%) had monolateral enlargement only. Pubic and/or axillary hair were not reported in any newborn.

### AGDs

Overall, in males both long and short AGDs were longer than in females as shown in **Table 2**. Percentiles of neonatal AGDs for males and females are shown in **Table 3**.

**Table 2:** AGD reference values for males and females at birth. Data are expressed as mean ± SD.

Long AGDs		Short AGDs		N	
AGDAC (mm)	AGDAP (mm)	AGDAF (mm)	AGDAS (mm)	F	M
38.5 ± 7.86	42.0 ± 7.21	14.1 ± 5.33	19.5 ± 8.30	196	214

**Table 3:** Percentile values of AGDs in newborn males and females. Data are expressed in mm.

Percentile	AGDAP (mm)	AGDAS (mm)	AGDAC (mm)	AGDAF (mm)
97th	60	36	55	26
95th	56	34	54	25
90th	54	31	51	23
75th	49	26	46	19
50th	43	20	38	14
25th	38	13	33	11
10th	33	10	30	9
5th	31	8	29	8
3rd	30	7	28	8

Considering the 5 subgroups according to GA, both long and short AGDs were longer in males compared to females at all gestational ages. AGD_AP_ was significantly longer in the newborns born at and after 40 weeks of GA (p<0.05) whereas AGD_AS_ was significantly longer in the males born at and after 39 weeks of GA (p<0.05).

In males, AGD_AS_ increased with increasing GA (from 15.8 mm in subgroup 1 up to 21.4 mm in subgroup 5).

Data are shown in **Tables 4**, **5**.

**Table 4:** AGD_AC_ and AGD_AP_ reference values in newborns according to GA. Data are expressed as mean ± SD.

GA (weeks)	AGDAC N	AGDAC Mean ± SD (mm)	AGDAP N	AGDAP Mean ± SD (mm)	F-value	P-value
37-37 + 6	4	N/A*	13	40.3 ± 5.71	χ2a=0.395	p=0.530
38-38 + 6	27	37.5 ± 7.32	15	41.9 ± 7.41	F1;40 = 3.43	p=0.071
39-39 + 6	44	39.4 ± 7.62	44	40.7± 7.60	F1;86 = 0.638	p=0.427
40-40 + 6	73	38.1 ± 8.59	68	42.3± 6.99	F1;139 = 9.99	p=0.002
≥41	42	38.0 ± 6.95	68	42.8± 7.37	F1;108 = 11.4	p=0.001

*Not reliable for scarce numerosity.

**Table 5:** AGD_AF_ and AGD_AS_ reference values in newborns according to GA. Data are expressed as mean ± SD.

GA (weeks)	AGDAF N	AGDAF Mean ± SD (mm)	AGDAS N	AGDAS Mean ± SD (mm)	F-value	P-value
37-37 + 6	4	N/A*	13	15.8 ± 6.38	χ2a=0.727	p=0.394
38-38 + 6	27	13.7 ± 4.89	15	15.9 ± 7.06	F1;40 = 1.50	p=0.227
39-39 + 6	44	14.3 ± 5.55	44	18.3± 8.53	F1;86 = 6.64	p=0.012
40-40 + 6	73	14.6 ± 5.54	68	20.1 ± 7.72	F1;139 = 24.0	p<0.001
≥41	42	14.3 ± 5.31	68	21.4 ± 8.89	F1;108 = 21.0	p<0.001

*Not reliable for scarce numerosity.

Considering AGDs according to BW, AGD_AP_ was significantly longer than AGD_AC_ in newborns with a BW between 3001 and 4000 g (p<0.05), whereas AGD_AS_ was significantly longer than AGD_AF_ in newborns with a BW between 2501 g and 4000 g (p<0.05), as shown in **Tables 6**, **7**.

**Table 6:** AGD_AC_ and AGD_AP_ reference values in newborns according to BW. Data are expressed as mean ± SD.

BW (g)	AGDAC N	AGDAC Mean ± SD (mm)	AGDAP N	AGDAP Mean ± SD (mm)	F-value	P-value
2501-3000	44	38.9 ± 7.97	26	41.4 ± 6.69	F1;68 = 1.79	p=0.185
3001-3500	85	37.4 ± 7.52	80	41.1 ± 7.09	F1;163 = 10.1	p=0.002
3501-4000	61	39.5 ± 8.30	80	42.9 ± 7.43	F1;139 = 6.41	p=0.012
≥4001	6	39.8 ± 7.25	26	42.7 ± 7.55	F1;30 = 21.4	p=0.413

**Table 7:** AGD_AF_ and AGD_AS_ reference values in newborns according to BW. Data are expressed as mean ± SD.

BW (g)	AGDAF N	AGDAF Mean ± SD (mm)	AGDAS N	AGDAS Mean ± SD (mm)	F-value	P-value
2501-3000	44	14.0 ± 5.55	26	17.7 ± 7.94	F1;68 = 5.20	p=0.026
3001-3500	85	14.4 ± 5.41	80	19.6 ± 7.22	F1;163 = 27.7	p<0.001
3501-4000	61	15.0 ± 5.18	80	20.8 ± 8.80	F1;139 = 21.2	p<0.001
≥4001	6	10.7 ± 2.42	26	17.3 ± 10.10	F1;30 = 21.4	p=0.123

### Penile length

The PL of male newborns (N = 208) was 24.4 ± 6.83 mm. Percentiles of PL are reported in **Table 8**.

**Table 8:** Percentiles of PL in male newborns.

Percentile	Penile length (mm)
97th	36
95th	35
90th	34
75th	30
50th	24
25th	20
10th	16
5th	13
3rd	12

Data are expressed in mm.

**Figure 3:**
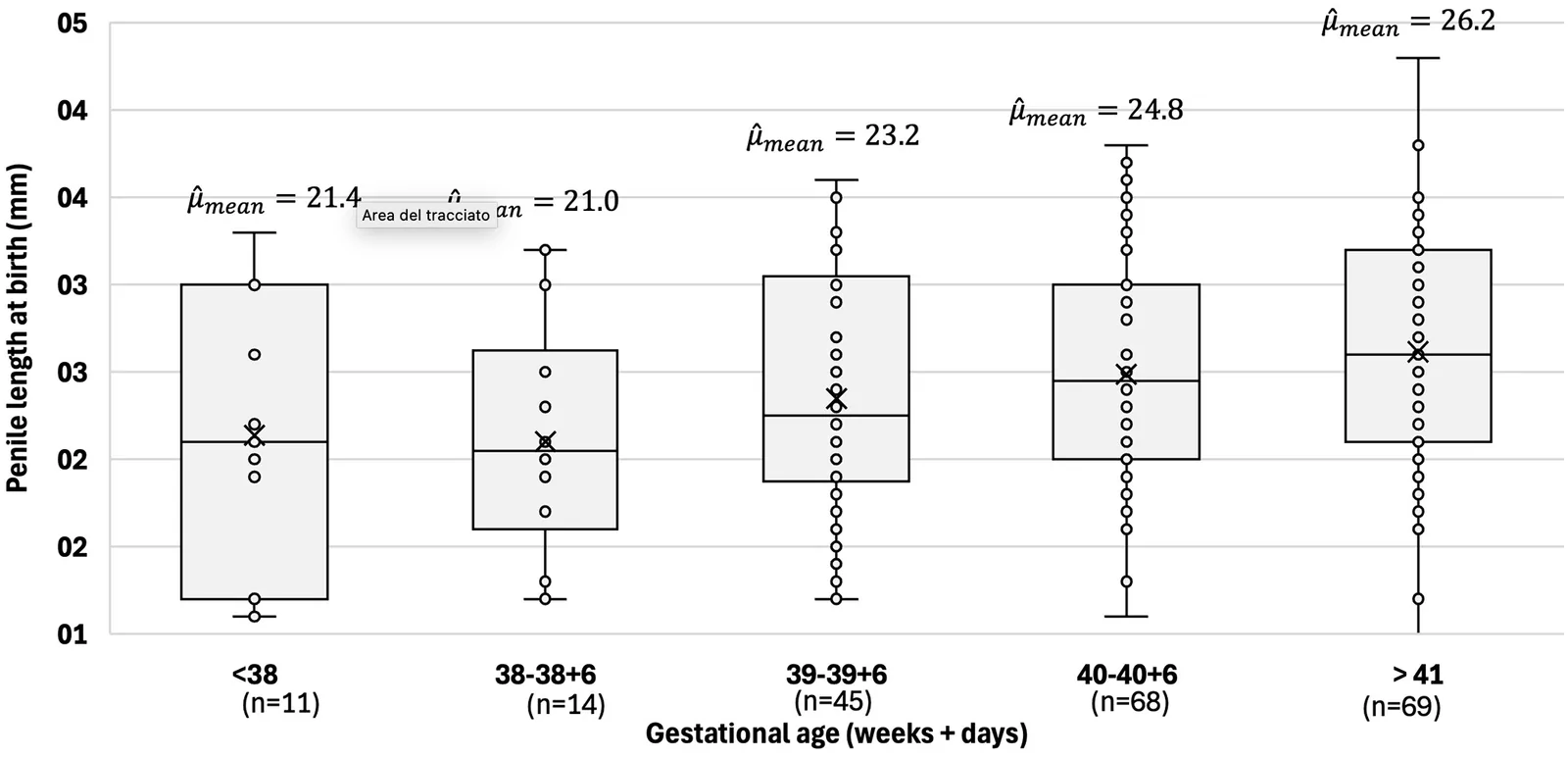
Mean, range and interquartile range (50% of sample) of PL (mm) at birth.

PL increased with GA as shown in **Figure 3**.

Considering PL according to BW, no clear trend was observed in relationship with BW (**Table 9**).

**Table 9:** PL reference values for male newborns according to BW. Data are expressed as mean ± SD.

Penile length of newborns according to BW		
BW (g)	N	Mean ± SD (mm)
2501-3000	23	21.3 ± 7.06
3001-3500	78	25.1 ± 6.75
3501-4000	84	24.9 ± 6.92
≥4001	23	23.5 ± 5.95

### Interexaminer variation

In males, interexaminer ICC values for penile length and both AGD_AS_ and AGD_AP_ were 0.945 (95% CI: 0.768-0.998), 0.905 (95% CI: 0.614-0.999), 0.902 (95% CI: 0.600-0.999), respectively. In females, interexaminer ICC values for AGD_AC_ and AGD_AF_ were 0.927 (95% CI: 0.705-0.998) and 0.831 (95% CI: 0.545-0.921), respectively.

### Correlation analysis of AGDs

In the Pearson correlation analysis, a positive significant correlation was found between AGD_AS_ and PL at birth (r=0.576, p<0.001) and between AGD_AS_ and GA (r=0.224, p<0.05). No other significant correlations were found among AGDs and neonatal and maternal features (**Table 10**).

**Table 10:** Pearson correlation analysis of maternal and newborn features associated with AGD in both sexes.

Features	AGDAP R-value	AGDAP P-value	AGDAS R-value	AGDAS P-value	AGDAC R-value	AGDAC P-value	AGDAF R-value	AGDAF P-value
Newborn features								
GA	0.097	0.972	0.224	0.013	-0.042	>.999	0.053	>.999
Weight	0.134	0.358	0.051	>.999	0.052	>.999	0.015	>.999
Length	0.051	>.999	0.009	>.999	-0.024	>.999	-0.039	>.999
Head circumference	-0.045	>.999	-0.020	>.999	-0.068	0.899	0.109	0.899
Weight-for length z-score	0.079	>.999	-0.070	>.999	0.083	0.250	0.044	>.999
Penile length	0.148	0.320	0.576	<0.001				
Maternal features								
Age	-0.007	>.999	-0.152	0.141	-0.049	>.999	-0.074	>.999
Weight before pregnancy	-0.047	>.999	0.003	>.999	-0.044	>.999	-0.034	>.999
Weight at the end of pregnancy	-0.039	>.999	0.021	>.999	-0.012	>.999	-0.005	>.999
BMI before pregnancy	-0.018	>.999	0.001	>.999	-0.009	>.999	-0.046	>.999

## Discussion

This cross-sectional study provides normative values for AGDs and PL according to GA and BW in healthy term newborns of both sexes. We have previously highlighted the role of AGD as indirect indicators of androgen exposure, its implication for human reproductive health outcomes and its association with exposure to EDCs (2).

Overall, both AGD_AP_ and AGD_AS_ were confirmed to be longer than AGD_AC_ and AGD_AF_ respectively, in agreement with previous studies (8, 9, 11, 13, 19, 20, 21). This difference in AGD measurements reflects the location of the caudal border of the genital swelling that during embryogenesis moves ventrally under the influence of androgens and differentiates into the labia majora in females and the scrotum in males (1, 13).

Previous studies reported a difference in sexes with AGD_AS_ being 1.66 to 2.5-fold longer compared to AGD_AF_ (5, 10, 22); this is slightly more than found in our study where AGD_AS_ was overall 1.4 times longer than AGD_AF_. This could be explained by environmental factors, ethnic reasons and different methods of measurement.

The AGD_AF_ mean value observed in our study (14.1 mm) was similar to that reported in the US, Thai, Indian, Spanish, Greek, Ghana and in one Italian study from the province of Modena (8, 13, 19, 20, 22, 23) and slightly higher than the value reported in UK, Mexico, India, China and Turkey (1, 5, 9, 10, 12, 21).

The AGD_AS_ mean value observed in our study (19.5 mm) was similar to the value reported previously in the Modena study, in UK, and Mexico (1, 9, 10, 13, 24) but shorter than that reported in US, Nigeria, Greece and Spain, Iran, Thailand, India, Korea, Ghana, China and Turkey (5, 8, 11, 19, 20–23, 25, 26).

The AGD_AC_ mean value in our study (38.5 mm) was similar to the value reported previously in Italy in a smaller cohort (13), lower than the value measured in Thailand (19), higher than that reported in Mexico, India, Greece, Ghana, Turkey, Spain and US (8, 9, 12, 20, 21, 23).

The AGD_AP_ mean value in our study (42 mm) was similar to the measurements reported in Korea (26), shorter than the values reported in all the other studies (8, 9, 11, 13, 19, 20, 21, 23, 24).

Mean values of AGDs and PL currently described in literature are provided in **Supplementary Table 1**.

The variability in AGD measurements and, thus, comparisons among AGD values in different countries, could be explained first of all by the different methods of measurement used (1, 8, 10, 24) and different positioning of the newborns. In fact, a systematic difference was described between the TIDES and Cambridge methods for AGD_AS_ and AGD_AF_ that were on average 12.9% and 7.4% longer using the TIDES versus the Cambridge method (6) due to a slightly greater stretch of the perineum (19). Differences among centers could also be due to differences in landmarks: Fisher and al. report better replicability of AGD_AS_ using the TIDES method, whereas AGD_AF_ and AGD_AP_ have poor replicability due to the difficulty to recognize distinct landmarks (6).

Furthermore, variability could be motivated by differences in the accuracy of tools utilized (23) and by the stretching of the perineum. Particularly in females, the swelling of the external genitalia that occurs in the first hours of life and the less distinct landmarks on soft tissues could affect AGD measurements (13). Ethnicity could also play an important role in determining variability of AGDs. Studies showed that maternal hormone levels during pregnancy might be different in different ethnic groups and may influence fetal hormone levels, affecting AGD and, thus, determining variability among different ethnic groups (5, 24, 27).

Considering AGD reference values based on GA and BW, these have been previously considered in a study on 1867 newborns (5), reporting findings similar to ours consisting in AGD_AS_ being 1.99-fold longer than AGD_AF_ and reporting a gradual increase of length with increasing GA and BW. This trend has been reported previously in the Literature and appears in contrast with testosterone serum concentration variations during gestation. Indeed, human male fetal Leydig cells start to produce testosterone at 8 weeks of gestation and levels peak between 11 and 14 weeks, decreasing steadily after 20 weeks of gestation. This suggests that AGDs are influenced by testosterone levels in early gestation but as GA advances, it becomes independent of the testosterone levels (2, 28).

The mean PL in our study was 24.3 mm, similar to the results reported in a smaller previous Italian cohort (13), shorter than the mean reported in the majority of other countries (2, 10, 11, 21–26, 29–32) and longer than that reported in China (5). This could probably depend on the ethnicity.

In our study, PL increased with GA as previously reported (5, 22); however, different from the Chinese study (5), PL did not seem to increase with BW, suggesting the need for normative data based on GA. The correlation we found between AGD_AS_ and PL at birth has been previously described (2, 10), and confirms that both are influenced by the same drivers (testosterone) during early gestation. Some studies have then shown that in contrast with AGD, PL continues to increase also in late gestation under the influence of testosterone suggesting that if a newborn has a normal AGD and micropenis, androgen insufficiency probably occurred after the MPW (2).

Interexaminer ICC values were high for PL and all AGDs both in males and females, showing excellent replicability for AGD_AS,_ AGD_AP_ and AGD_AC_ and good replicability for AGD_AF._ Previous studies reported lower interexaminer ICCs for AGDs than those observed in our study; only AGD_AS_ showed better replicability with the TIDES method, probably due to the difficulties identifying landmarks for the other AGDs (6). The higher reproducibility observed in our study may be attributable to the standardized measurement method shared among the examiners.

Different studies have reported associations of AGDs with newborn clinical features such as GA, BW, birth length and head circumference and maternal factors (5, 8–12, 23). In particular, some studies identified BW as strong determinant of AGDs (5, 20, 22) and some authors suggested that adjusting AGDs for length may be a more appropriate way to account for body size than using weight, as the latter could be influenced by exogenous factors as nutrition, parity, pregnancy-related overweight or environmental exposure (9). Our results showed only a mild correlation between AGD_AS_ and GA and no other significant correlations among AGDs and neonatal and maternal clinical variables; therefore, further studies are needed to clarify whether adjustment for these factors is warranted.

Our study has strengths and limitations. One of the main strengths is represented by the large cohort, furthermore, a well-reproducible and standardized method (6) shared among the professionals to improve the reliability of the measurements. Mother-infant dyads represented a selected cohort: mothers were healthy and pregnancies uncomplicated, newborns were at term and all appropriate for gestational age. Variable sample sizes across analyses resulted from missing measurements, mainly due to maternal unavailability to participate and other logistical constraints. Furthermore, the sample size of neonates with GA under 39 weeks should be considered when interpreting the subgroups analyses. We did not collect blood or urinary samples for hormonal dosages because this was not the purpose of the LIFE- MILCH project, but further studies could be useful to investigate the relationships among AGD, penile length, puberal stages and hormonal levels. A limitation of this study is that percentiles were derived empirically rather than using advanced centile modelling techniques. Due to limited sample sizes in some GA and BW subgroups, the results should be considered descriptive reference values rather than fully modelled growth standards. Future large-scale studies are warranted to establish smoothed centile curves using advanced reference-modelling approaches.

In conclusion, this study provided measurements and reference values for AGDs and PL, also according to GA and BW in a large group of newborns, providing a valid well-reproducible instrument for clinical practice and the basis for further studies, particularly those related with the emerging effects of endocrine disruptors on reproductive health.

## Data Availability

The original contributions presented in the study are included in the article/**Supplementary Material**. Further inquiries can be directed to the corresponding author.
