# Supplementary material for: External genital features and normative values for anogenital distances and penile length at birth in a healthy Italian population: data from the LIFE-MILCH project
Source: Front Endocrinol (Lausanne). 2026 Aug 6;17:1838976. doi: 10.3389/fendo.2026.1838976 (PMC13489917; doi:10.3389/fendo.2026.1838976)
Supplement: Supplementary file 1 [file Table1.docx]

Supplementary Material.

# 1 Supplementary Table

Supplementary Table S1. Mean values of AGDs and PL currently described in literature. Data are expressed as mean ± SD.

|  | **Long AGDs** | | **Short AGDs** | | **PL (mm)** |
| --- | --- | --- | --- | --- | --- |
| **Reference** | **AGD_AC_ (mm)** | **AGD_AP_ (mm)** | **AGD_AF_ (mm)** | **AGD_AS_ (mm)** |  |
| Our study | 38.5 ± 7.9 | 42.0 ± 7.2 | 14.1 ± 5.3 | 19.5 ± 8.3 | 24.4 ±6.8 |
| 1 | / | / | 11 ± 2 | 21 ± 3 |  |
| 2 ^a^ |  |  |  |  | 30.38 ± 4.1 |
| 5 | / | / | 11.65 ±1.5 | 23.18 ± 3.2 | 18.90 ± 3.0 |
| 8 | 36.7 ± 3.9 | 49.6 ± 5.9 | 16.0 ± 3.2 | 24.7 ± 4.5 | / |
| 9 | 35.9 ± 5.7 | 49.3 ± 5.8 | 10.2 ± 1.2 | 19.1 ± 3.7 |  |
| 10 | / | / | 9.1 ± 2.8 | 19.8 ± 6.1 | 30.4 ± 4.3 |
| 11 | / | 48.7 ± 3.9 | / | 25.5 ± 3.9 | 39.4 ± 4.2 |
| 12 | 31.8 ± 3 | / | 9.7 ± 1.7 | / | / |
| 13 | 38.6 ± 7.1 | 48.8 ± 5.8 | 12.0 ± 4.3 | 19.6 ± 6.6 | 24.3 ± 5.9 |
| 19 | 42.6 ± 6.2 | 52.6 ± 6.9 | 16.5 ± 3.9 | 25.2 ± 4.8 |  |
| 20 | 34.9 ± 3.1 | 48.5 ± 5.2 | 14.1 ± 2.8 | 25.2 ± 5.1 | / |
| 21 | 30.0 ± 2.0 | 56.0 ± 1.0 | 10.3 ± 2 | 23.0 ± 0.6 | 32.0 ± 2.0 |
| 22 | / | / | 15.5 ± 1.6 | 25.7 ± 3.0 | 33.2 ± 3.8 |
| 23 | 34.2 ± 3.3 | 48.9 ± 5.6 | 13.6 ±2.7 | 25.5 ± 5.0 | 33.0 ± 0.5 |
| 24 ^b^ | / | 49.9(38.4-74.7) | / | 19.1 (8.7-35.8) | 27.1(15.5-42.9) |
| 25 | / | / | / | 24.4 ± 2.5 | 32.1 ± 3.5 |
| 26 | / | 42.0 ± 3.0 | / | 23 ± 2.0 | 33.0 ± 2.0 |
| 29 |  |  |  |  | 35.0 ± 4.0 |
| 30 |  |  |  |  | 35.5 ± 5.7 |
| 31 |  |  |  |  | 35.0 ± 7.0 |
| 32 |  |  |  |  | 35.7 ± 4.6 |

^a^AGD not comparable because preterm included (GA 22-41 weeks). ^b^ Data are expressed in median (min-max).
